# The Study on Stage Financing Model of IT Project Investment

**DOI:** 10.1155/2014/321710

**Published:** 2014-07-23

**Authors:** Si-hua Chen, Sheng-hua Xu, Changhoon Lee, Neal N. Xiong, Wei He

**Affiliations:** ^1^Institute of Information Resource Management, School of Information Technology, Jiangxi University of Finance and Economics, No. 169, East Shuanggang Road, Changbei, Nanchang, Jiangxi 330013, China; ^2^Department of Computer Science and Engineering, Seoul National University of Science and Technology, Seoul 139-743, Republic of Korea; ^3^School of Business and Administration, Jiangxi University of Finance and Economics, No. 169, East Shuanggang Road, Changbei, Nanchang, Jiangxi 330013, China

## Abstract

Stage financing is the basic operation of venture capital investment. In investment, usually venture capitalists use different strategies to obtain the maximum returns. Due to its advantages to reduce the information asymmetry and agency cost, stage financing is widely used by venture capitalists. Although considerable attentions are devoted to stage financing, very little is known about the risk aversion strategies of IT projects. This paper mainly addresses the problem of risk aversion of venture capital investment in IT projects. Based on the analysis of characteristics of venture capital investment of IT projects, this paper introduces a real option pricing model to measure the value brought by the stage financing strategy and design a risk aversion model for IT projects. Because real option pricing method regards investment activity as contingent decision, it helps to make judgment on the management flexibility of IT projects and then make a more reasonable evaluation about the IT programs. Lastly by being applied to a real case, it further illustrates the effectiveness and feasibility of the model.

## 1. Introduction

For the private enterprises in early stage or expansion stage, if they have advantages in technologies, products, markets, or teams and possess the capability of fast growth, they may attract the attraction of venture capital. However, due to the enormous uncertainties faced by these enterprises, there are high risks for the venture capital to invest in these enterprises (we call them venture enterprises). Therefore, the investment to venture enterprises always takes the form of stage financing. That is, venture capital investors usually do not invest all to the venture enterprise at one time. They usually invest part of the capital of the enterprise at each development stage and remain the right to give up investment and to liquidate of any stage. The stage financing depends on the degree of information symmetry, the degree of satisfaction to the need of information, capital structure, and requirements of management incentive [1–4]. Stage financing is a kind of motivation method to venture enterprises [5–7]. There are two types of stage financing: milestone investment and round investment. For the first type, both sides set the goal of each stage and decide the total investment in the first contract to avoid negotiating again. In this way, venture capital investors can get high flexibility in advance. Once the venture entrepreneur cannot achieve the set goals (such as obtaining new patents, producing final products, or getting the foreseeable product market sales), venture capital investors have the right to give up the project. For round investment, it offers venture capital investors great flexibility afterwards. Usually they do not decide the total investment in the first contract. After each stage of investment, both sides need to negotiate about the investment of next stage. According to the theory of stage financing, scholars agree that stage financing can reduce risk of investment [8, 9], effectively motivate venture entrepreneurs [10], and reduce the commitment problem of renegotiation of venture entrepreneurs [11, 12].

## 2. Related Works

Stage financing depends on factors such as the extent of information symmetry, the extent of satisfaction to the need of information, capital structure of enterprises, and management incentives. Admati and Pfleiderer showed that entrepreneurs had typical investment preferences. As long as there is somebody willing to invest, the entrepreneurs will never give up the business he starts no matter how it is clear that the business will be a failure [13]. Before the investment, it is probable that the investors may not know the information of the business. Therefore, the full and one-off capital investment may bear high agent risk. According to traditional principal-agent theory, the asymmetry of information between the principal and the agent will result in the violation behaviors of the agent. In venture capital investment, due to the participation of risk capitalists, the asymmetry of information has been greatly reduced to large degree but this phenomenon still exists. Triant (2001) held the view that because information could not be effectively transferred to the investors, the discrimination ability of the investors on entrepreneurs and their investment project was weakened. Such kind of asymmetry of information not only increases the investment risk but also increases the financing cost of entrepreneurs [14]. The empirical study of Cumming and MacIntosh (2001) indicated that there was asymmetry of information in venture capital investment especially in early stage [15]. Adopting stage financing can reduce the extent of asymmetry of information. The extent of satisfaction to the need of information can influence stage financing from another perspective. Generally speaking, the higher the extent of satisfaction of information needs is, the less the stages of venture capital investment are. K. Smith and R. L. Smith (2000) pointed out that entrepreneurs knew better about the advantages of their technology while investors might know more about the economic values of project [16]. The information for investors to evaluate the investment opportunity is asymmetric and highly uncertain. By stage financing, the investors can obtain both internal and external information of enterprises at different stages and reduce investment risks. In addition, the capital structure of enterprises also influences stage financing. Gompers's study showed that when the ratio of intangible capital of risk enterprises is high, the agency cost and supervision cost are high and the stages of stage financing are more [17]. That is, the stages of stage financing are positively proportional to the ratio of intangible capital. A distinct feature of venture capital investment is management participation. By a series of institutions, an effective governance mechanism is formed which can promote the effective operation of risk enterprises and reduce management risks. Management incentives are the basic governance for risk enterprises. Cornelli and Yosha (1998), Gompers (1995), Sahlman (1990) pointed out the rights that investors could give up the investment liquidate at any stage are not only the pressures on the management of risk enterprises but also the incentives on the management. Therefore, stage financing is a kind of incentive method to risk enterprises [5, 18–25].

Stage financing is the basic strategy for venture capital investment. The process of stage financing is accompanied with stage evaluation. In fact, before each investment investors need to evaluate risk enterprises. It is recognized that the evaluation on risk enterprises consists of two perspectives: one is nonvalue evaluation on risk enterprises, that is, based on reasonable evaluation indicator system to make a comprehensive evaluation on the development capability of risk enterprises. Another is value evaluation, that is, to make an evaluation on the value of equity or value of risk enterprises [26–30]. From the perspective of investors, no matter what kind of form we take to evaluate at any stage, the final goal is to maximize the value of equity or value of enterprises.

The study on the risk of IT project investment becomes one hot topic in present MIS field. In the development of IT project, it is very important to effectively identify all the involved risk factors and offer effective risk management measures [31–35]. It is usually thought that there are two ways to control risks: decreasing the probability of risks or reducing the significant outcome brought by risk. In the present studies and practices, people usually prefer to curbing risk by the ways of reducing the production scale, continuing to do R&D or cancelling the project to achieve the goal of reducing risk probability [36–38].

However, the happening of some risks cannot be eliminated. From economy aspect, it is not worthwhile to eliminate some risks. Therefore, we should comprehensively consider the methods of avoiding or transferring risks. However, the present studies on risk management of IT projects are not enough. Originated from finance, the real option tool which is widely used in financial risk management provides powerful tool for it.

## 3. Stage Financing and Hypothesis

### 3.1. Different Development Stages of Enterprises and Stage Financing

Generally, the development of venture enterprises always experiences five phrases including the R&D stage, the initial stage, the early growth stage, the rapid growth stage, and the withdrawing stage. The investment stage of venture investment is consistent with the development stage of venture enterprises but sometimes there is also inconsistency. In each investment stage, the new internal and external information about the enterprise is released and they are the important proof for whether to continue to invest.

The investors adopt “wait and see” way and obtain the specific business performance indicators milestones, such as analyzing tests, product prototype, the first production, and the first marketing. “Milestones” can work as the benchmarks for investment stages. Through business process, the investors continuously look for milestones. On the basis, they will constantly evaluate the value of enterprises and decide whether to further invest or cooperate.

### 3.2. The Thought of Stage Financing of IT Project

**Figure 1:**
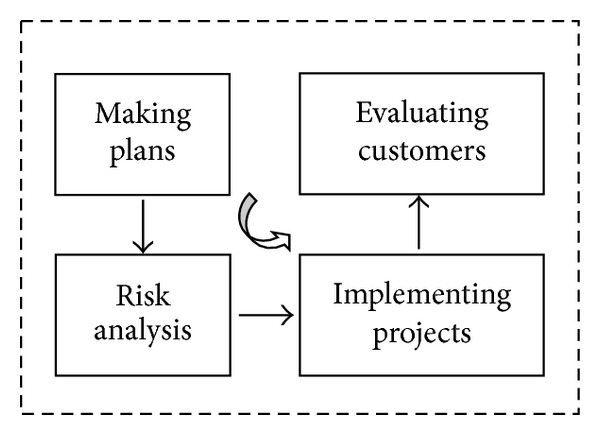
Multistage software development model.

In fact, the thought of stage financing of IT project has been adopted in the development of software program. In software engineering, the BOEHM spiral model is in essence a multistage software development model. The spiral model combines the waterfall model and rapid prototype model. The model supposes that the development of software programs is a spiral rising process with several iterative cycles. In each stage the following activities are carried out as shown in Figure 1:

1. making plans: defining the target of software and making plan, making clear the restrictive conditions of project development,
2. risk analysis: analyzing and evaluating the scheme and considering how to recognize and eliminate risks,
3. implementing projects: implementing software development and testing,
4. evaluating customers: evaluating development work and proposing revision suggestions and making next plan.

First of all, each stage should define the goals of this stage, the scheme to achieve these goals, and the restrictive conditions. Then we analyze the development strategy of each scheme from the aspect of risk and try to exclude all kinds of potential risks. Sometimes it is even needed to construct prototype to finish. If some risks cannot be excluded, the scheme should stop immediately otherwise we need to start next development step. Lastly we need to evaluate the outcome of this stage and design for the next stage. With the successful application of the thought of stage financing of IT program to software project, more and more venture investment of IT program adopts the decision of multistage investment thought to reduce the risks and cost of investment to obtain more profits.

## 4. Real Option Pricing Model

Suppose there exists a new venture investment project. The factors influencing the value of the project include the quality of venture entrepreneur, market needs, and progress level of technology. To consider all the factors, this paper supposes that the value of venture investment project obeys the geometric Brownian movement in two continuous time slots:

(1) $$\begin{array}{c} dV=\alpha Vdt+\sigma tVdW, \end{array}$$

where *V* means value and *t* means time. *α* is the drift coefficient, which is decided by the quality of venture entrepreneur and the conditions of total market. *σ* is variance coefficient; *dW* is a standard Wiener process. To simplify it, this paper supposes that *α* is a constant. There are three investment times: *T*_1_, and *T*_2_, 0 < *T*_1_ < *T*_2_. Suppose, at the time of *t* = *T*_2_, the venture investment project needs the last venture investment *M* and then the venture investment organization can carry out the withdrawing mechanism and obtain the benefits. We further suppose that, at the time of *t* = 0, there is no capital for venture enterprise and it needs to get venture investment to support the rapid development of the enterprise within the period of 0 < *t* < *T*_2_.

This paper makes a comparison and we suppose that the two venture investment schemes are as follows:

1. the stage financing: at the point of *t* = 0, the investment is *I*_1_; at the point of *t* = *T*_1_, the investment is *K*;
2. the single stage of investment: at the point of *t* = 0, we invest *I*_2_, *I*_2_ = *I*_1_ + *e* − *rT*_1_ *K* (*r* is risk free interest rate).

Because it is hard for venture enterprises to get other capital except for venture investment, this paper believes that if venture investment organizations do not invest, the value of venture enterprises will be 0. The agency cost is the cost to reconciliate the different goals of them. For the venture investment organization, its goal is to maximize the benefits of the venture investment project. And the option value of it is the main part of the benefits of venture investment.

Because of the characteristics of option, the value of option, and the value of venture investment project is positive to the risk level of the investment period *σ*. Therefore, the venture investment organization hopes that the venture entrepreneur choose the higher risk level. However, for venture entrepreneur, his goal is to maximize the possibility of obtaining next venture investment. Therefore, to ensure to achieve the goal of each stage, generally he will adopt conservative operation behavior so that the risk level is low. The inconsistency of goals will produce the agency cost. This paper quantitatively describes the agency cost as the degree of risk of which the venture entrepreneur can choose the operation behavior, that is, the value of *σ*.

In the single investment stage, because there is no the benefit brought by the reduced agency cost of the stage financing, the risk level chosen by venture entrepreneur will be lower than the risk level chosen in the stage financing. To simplify it, this paper supposes that once the risk level of the investment period is set, it will be constant.

For the stage financing, at the point of *t* = 0, after we invest *I*_1_, the venture investment organization will get a compound call option, that is, obtaining two rounds of investment choices: when *t* = *T*_1_, we invest *K*; when *t* = *T*_2_, we invest *M*. Geske (1979) once induced the pricing model:

(2) C1=VN(h1+σ1τ1,h2+σ2τ2,τ1τ2)−Me−rτ2N(h1,h2,τ1τ2)−Me−rτ1N(h1),

where

(3) h1=ln⁡(V/M)+(r−(1/2)σ12)τ1σ1τ1,h2=ln⁡(V/M)+(r−(1/2)σ22)τ2σ2τ2.

We can get the value of *V* from the formula:

(4) $$\begin{array}{c} VN\left(h_{2}+\sigma_{1}\sqrt{\tau }\right)-Me^{-r\tau }N\left(h_{2}\right)-K=0. \end{array}$$

*r* is risk free interest rate:

(5) $$\begin{array}{c} \tau_{1}=T_{1}-t;\;\;\tau =T_{2}-T_{1};\;\;\tau_{2}=T_{2}-t=\tau_{1}+\tau . \end{array}$$

*N* is cumulative standard normal distribution function, *N*(*A*, *B*, *ρ*) is bivariate cumulative standard normal distribution function, *A* and *B* are the upper limit of integral, and *ρ* is correlation coefficient. When *t* = 0, we can get

(6) $$\begin{array}{c} NV_{1}=C_{1}-I_{1}. \end{array}$$

For single stage investment, at the point of *t* = 0, after investing *I*_2_, we can get a simple call option. We can apply B-S model to calculate the value of the option:

(7) C2=VN(d1)−Me−r(T2−t)N(d2),d1=ln⁡(V/M)+(r+(1/2)σ12)(T1−t)σ1((T1−t)),d2=ln⁡(V/M)+(r+(1/2)σ22)(T2−t)σ2((T2−t)).

*r* is risk free interest rate; *N*(·) is cumulative standard normal distribution function. Then at the point of *t* = 0, we can get

(8) $$\begin{array}{c} NV_{2}=C_{2}-I_{2}. \end{array}$$

We compare the effect produced from stage financing with the single stage investment, that is, the odds between the net value of stage financing and the net value of single stage investment (*NV*_1_ − *NV*_2_). To further explore the contribution of stage financing, by using the difference analysis method, we define the difference of investment value due to different risk level at the same investment stage as risk effect; we define the effect produced by delaying some initial investment capital *K* to invest at the time of *t* = *T*_1_ as delay effect. The sum of delay effect and risk effect should be equal to the total effect of stage financing. The real option pricing method regards investment activity as contingent decision. Therefore, it can make judgment on the value of the management flexibility of the project and then make a more reasonable evaluation about the IT program.

## 5. The Real Option Analysis of Risk Aversion Strategy of Multistage

An enterprise is considering to develop a ERP system to integrate the internal business process of the enterprise. Once being established, the system will support the daily activities of the enterprise such as purchasing, selling, and production. The system is planned to be finished within 3 years and is estimated to be invested 250 thousand dollars. If it is successfully implemented, it will bring 300 thousand dollars profits. First, we adopt NPV (net value) method to evaluate the economic benefits of the project. We suppose the risk free interest rate is 12%. The expected earnings are 34 884 dollars.

When adopting NPV method, in fact they regard that the investment to the project is one-off. However, with the real option thought, we can apply stage financing strategy to avoid the risks of the project. For example, the storage management subsystem is an important part of ERP system. It is highly connected with the purchasing, selling, and logistics of the enterprise. Therefore, the decision maker can divide the ERP system into 2 phrases to develop. At the first stage, we can develop the storage management system. After the system is successfully implemented, the development team can go into the second stage to finish the development of other parts of the whole ERP system.

To simplify it, we suppose that the time of the first stage is one year. The development fees for storage management system are 50 thousand dollars. The time of the second stage is two years and the development cost is 200 thousand dollars. The following thing is to adopt the real option pricing method to evaluate the revised development strategy of the project to test the economic benefits of the strategy. The development cost of storage management system (*k* = 50 thousand dollars) can be regarded as a European call option of the investment of the second stage. The expected earnings of the option are *V* = 300 thousand dollars; the implementing cost of the option is *M* = 200 thousand dollars. Then we can apply Black-Scholes pricing model to calculate the option value *C*_0_:

(9) $$\begin{array}{c} C_{0}=VN\left(d_{1}\right)-Me^{-rT}N\left(d_{2}\right), \end{array}$$

where

(10) d1=ln⁡(V/M)+(1+(1/2)σ2)TσT,d2=d1−σT.

*σ* is the variance of expected earnings and we set it as 50%; *r* is the risk free interest rate; *N*(·) stands for cumulative normal distribution function. Taking the values of these parameters to the formula, we can get the option price: *C*_0_ = 162 559 dollars. Considering the development cost of the first stage, according to multistage strategy the earnings to develop the ERP project are 118 213 dollars. In another word, the multistage development strategy will bring 118 213 − 34 884 = 83 329 dollars extra earnings. At the same time, this project can also be regarded as a two-stage IT investment project. When the enterprise invests in the first stage, it also gets the investment option of the second stage. The compound option pricing formula proposed by [39] can precisely evaluate the compound option. So here this paper will use this compound option pricing formula to estimate the value of the project again. The pricing formula is as follows:

(11) C1=VN(h1+σ1τ1,h2+σ2τ2,τ1τ2)−Me−rτ2N(h1,h2,τ1τ2)−Me−rτ1N(h1),

where

(12) h1=ln⁡(V/V−)+(r−(1/2)σ12)τ1σ1τ1,h2=ln⁡(V/M−)+(r−(1/2)σ22)τ2σ2τ2.

From formula $VN(h_{2}+\sigma_{1}\sqrt{\tau })-Me^{-r\tau }N(h_{2})-K=0$, we can get the value of *V*. Making *V* = 300000 dollars, *K* = 50000 dollars, *M* = 200000 dollars, *T*_1_ = 1 year, *T*_2_ = 3 years, *r* = 12%, *σ* = 50%, we can get the value of compound option *C*_0_ = 134 659 dollars. Comparing the value of compound option with net present value, the value of option of the project increases 134 659 − 34 884 = 99 775 dollars.

## 6. Conclusions

The IT projects always have high risks. The benefits of projects are influenced by many uncertain factors. Some studies pointed out that the traditional NPV method may underestimate the value of project in uncertain environment. Sometimes the value may be underestimated about half of it. Dividing the development of ERP system into 2 stages is in fact providing a waiting option for decision makers. If the development of first stage is successful and can achieve good effect, then we can continue to develop other parts of ERP system. On the contrary, if the development of first stage fails or there appear some disadvantageous factors, the decision makers may delay the development of the second stage or even cancel the whole project to avoid larger loss.

The wrong need analysis or unclear need definition is one of the most common risks in the development of IT projects. Because there is high correlation between storage management system and other parts of ERP system, the development of first stage will help development staff to understand the business process of the enterprise and better understand and design customers' needs so as to better reduce the need risks of the second stage and increase the success probability of the project.

For some IT projects such as big ERP projects, they are generally highly complex. In particular, when it adopts new technology or has lots of interactions with external systems, the development task is more difficult. The strategy of stage financing is equal to first making experiment on small scale which provides the learning opportunity for development staff to get familiar with new technologies. It also can reveal the potential problems and then help to make corresponding measures, such as developing some assisting tools to reduce the complexity risks of the project.

This paper applies the real option pricing model to measure the value brought by the stage financing strategy. But there are still some limitations. First of all, the factors which influence investment such as management participation and incentives have not been included in this model. Secondly, this paper only considers the value of equity and has not considered the application of other financial tools such as convertible bonds, preferred stock to stage financing. All these problems still wait to be explored in future research.
